# The Inhibition of Stat5 by a Peptide Aptamer Ligand Specific for the DNA Binding Domain Prevents Target Gene Transactivation and the Growth of Breast and Prostate Tumor Cells

**DOI:** 10.3390/ph6080960

**Published:** 2013-08-20

**Authors:** Axel Weber, Corina Borghouts, Christian Brendel, Richard Moriggl, Natalia Delis, Boris Brill, Vida Vafaizadeh, Bernd Groner

**Affiliations:** 1 Georg-Speyer-Haus, Institute for Biomedical Research, Frankfurt am Main 60596, Germany; E-Mails: axel.weber@em.uni-frankfurt.de (A.W.); delis@em.uni-frankfurt.de (N.D.); brill@em.uni-frankfurt.de (B.B.); v.vafaizadeh@med.uni-frankfurt.de (V.V.); 2 Ganymed Pharmaceuticals AG, Mainz 55131, Germany; E-Mail: c.heinz@ganymed.ag; 3 Boston Children’s Hospital, Division of Hematology/Oncology, Boston MA 02115, USA; E-Mail: christian.brendel@childrens.harvard.edu; 4 Ludwig Boltzmann Institute for Cancer Research (LBI-CR), Vienna 1090, Austria; E-Mail: richard.moriggl@lbicr.lbg.ac.at

**Keywords:** peptide aptamer (PA), signal transducer and activator of transcription 5 (Stat5), RNA interference (RNAi), yeast-two-hybrid (Y2H), protein transduction, prostate and breast cancer cell lines, inhibition of DNA-binding

## Abstract

The signal transducer and activator of transcription Stat5 is transiently activated by growth factor and cytokine signals in normal cells, but its persistent activation has been observed in a wide range of human tumors. Aberrant Stat5 activity was initially observed in leukemias, but subsequently also found in carcinomas. We investigated the importance of Stat5 in human tumor cell lines. shRNA mediated downregulation of Stat5 revealed the dependence of prostate and breast cancer cells on the expression of this transcription factor. We extended these inhibition studies and derived a peptide aptamer (PA) ligand, which directly interacts with the DNA-binding domain of Stat5 in a yeast-two-hybrid screen. The Stat5 specific PA sequence is embedded in a thioredoxin (hTRX) scaffold protein. The resulting recombinant protein S5-DBD-PA was expressed in bacteria, purified and introduced into tumor cells by protein transduction. Alternatively, S5-DBD-PA was expressed in the tumor cells after infection with a S5-DBD-PA encoding gene transfer vector. Both strategies impaired the DNA-binding ability of Stat5, suppressed Stat5 dependent transactivation and caused its intracellular degradation. Our experiments describe a peptide based inhibitor of Stat5 protein activity which can serve as a lead for the development of a clinically useful compound for cancer treatment.

## 1. Introduction

The Jak-Stat signaling pathway is an essential cellular communication route from the plasma membrane to the nucleus. It can be activated by cytokines, hormones and growth factors and is involved in the regulation of homeostasis and tissue-specific functions [1,2]. In mammals the Stat protein family consists of seven members (Stat1, 2, 3, 4, 5a, 5b and 6) with similar domain structures and lengths ranging from 750 to 850 amino acids. They comprise an amino terminal tetramerization domain, followed by a coiled-coil and central DNA-binding domain (DBD), a linker domain, a characteristic SH2-domain and a carboxyl terminal transactivation domain. The TAD determines the tissue and cell type specific activity of Stats. Its function is modulated by secondary modifications and cofactor interactions. Activation of Stats occurs via phosphorylation of a tyrosine residue located between the SH2- and TAD-domains. The activating kinases comprise receptor associated Janus family kinases (Jak), receptor tyrosine kinases, e.g., EGFR or cytoplasmic tyrosine kinases, e.g., Src. Stat proteins form transactivating dimers of parallel conformation by reciprocal interaction of their SH2 and their tyrosine phosphorylated domains [3]. This conformation exposes a nuclear localization signal (NLS) within the DBD of Stat dimers [4], which allows them to bind to importin-α and translocate into the nucleus. There they bind to consensus GAS-motifs, gamma interferon activated site: TTCNNNGAA, present in target gene promoters. Additional non-canonical functions, functions not dependent upon the transcriptional transactivation of genes, have recently been described for members of the Stat family. Phosphorylated dimers or non-phosphorylated monomers of Stat3 and Stat5 can assume additional functions in various subcellular compartments. These functions regulate the maintenance and modification of mitochondria, the Golgi apparatus and rough endoplasmatic reticulum, the modification of heterochromatin and the cytoskeleton as well as cofactor activities [1,5,6].

The isoforms Stat5a and Stat5b share 95% sequence identity at the protein level. Homo- and heterodimer can be formed. They were originally identified as transcription factors of milk protein genes [7,8], but many additional Stat5-mediated functions have since been discovered. Both genes are ubiquitously expressed, and 3,000 to 100,000 molecules have been detected per cell, depending on the cell type and differentiation state [9,10]. Stat5 is a master regulator of hematopoiesis and mammogenesis and also exerts a strong impact on immune, prostate and liver cell functions. The activation of Stat5 is linked to malignant transformation in these tissues. The deregulation of the extent and the duration of Stat3 and Stat5 activation is an important contributor to tumorigenesis. Persistently activated Stat3 and Stat5 molecules and Stat induced target gene expression can be found in tumor cells. They can regulate hallmarks of cancer [11] and cause increased proliferation, survival, immune evasion, angiogenesis, migration and metastasis or reprogramming of energy metabolism [12,13]. The survival of tumor cells can become dependent on the continuous activity of Stat3 or Stat5. Tumor cells react to their inhibition with induction of apoptosis. Both, Stat3 and Stat5, represent promising molecules in targeted cancer therapy. In hematological malignancies, the constitutive Stat5 activity, induced by leukemic oncogenes like Bcr-Abl, is associated with tumor maintenance by regulating survival, self-renewal and expansion of leukemic stem- and progenitor cells [14,15,16]. In solid tumors the deregulation of Stat5 can be a consequence of an excess of activating ligands in the tumor microenvironment, but also a consequence of the overexpression or mutation of cognate receptors. However, aberrant Stat5 activation does not equally affect the phenotypes of tumors of different origins. In prostate cancers, constitutive Stat5 activity is associated with the development of aggressive, fast proliferating and invasive phenotypes. The activity of Stat5 in breast cancers is correlated with more differentiated and less invasive phenotypes and a more favorable clinical outcome [17].

The transforming potential of persistently activated Stat5 is not limited to particular indications. We evaluated Stat5 expression and activity in solid tumor cells of different origins. Four human tumor cell lines, derived from breast (T-47D), prostate (PC-3), epidermal (A431) and colorectal (HCT116) cancers, were employed and their response to the inhibition of Stat5 was investigated. Two approaches to the inhibition of Stat5 were used. RNA interference was used to affect the stability and the translation of Stat5 specific mRNA and cause the downregulation of its expression. shRNAs directed against both isoforms of Stat5 were derived. The effects of Stat5 inhibition due to downregulation of mRNA were compared to the effects elicited by an inhibitor of the DNA-binding function of Stat5. This was achieved by a peptide aptamer (PA) ligand which specifically binds to the DBD of Stat5 (S5-DBD-PA: Stat5-DNA-binding domain specific peptide aptamer).

We employed a peptide-based ligand of Stat5 as a specific inhibitor, because Stat5 lacks hydrophobic binding pockets, which usually characterize conventional drug targets and which are necessary to identify effective inhibitors of low molecular weight. Proteins often interact with each other through flat surfaces and their interaction is not necessarily dependent on a presence of hydrophobic pocket structures [18]. PA can therefore be exploited as specific ligands and potential inhibitors. They are able to bind to any pre-determined target domain and can be developed into therapeutic agents. They possibly can inhibit a broad spectrum of intracellular proteins and thus extend the range of conventional drug targets. Yeast-two-hybrid screens with synthetic and random 12mer peptides as prey constructs have been used to identify a PA sequence, which binds to the DNA-binding domain of Stat5 (Stat5-DBD) with high specificity and affinity. The PA sequence has been displayed in a constrained and unique conformation on the surface of a stable scaffold protein, by introduction in the protruding active site of a modified version of the human thioredoxin protein (hTRX). Additional functional domains were added which optimized the expression of the recombinant S5-DBD-PA protein construct, allowed for its purification and its delivery into target cells by protein transduction [19]. Complex formation of the Stat5-DBD with S5-DBD-PA led to the inhibition of the DNA binding capacity of Stat5, prevented nuclear translocation of Stat5 dimers and induced its proteasomal degradation. The expression of Stat5-shRNA or S5-DBD-PA upon lentiviral gene transfer strongly affected tumor cell viability. The same was observed upon uptake of recombinant S5-DBD-PA protein added to the cell culture medium. HCT116 colorectal carcinoma cells served as controls, these cells express only very low levels of Stat5 and are not dependent on Stat5 functions. However, the cell lines A431 and T-47D also express relatively low levels of tyrosine phosphorylated Stat5. Nevertheless, survival of these cells seems to be sensitive to Stat5 downregulation, an effect possibly due to the inhibition of the non-transcriptional functions of Stat5. Therapeutic agents able to discriminate between the individual functions of Stat5 in different subcellular compartments might become desirable for effective tumor therapy and the prevention of side effects in normal tissues.

## 2. Results and Discussion

### 2.1. RNA Interference Validates Stat5 as a Therapeutic Target in Breast, Prostate and Epidermoid Carcinoma Cells

Stat5 activation has been shown to strongly influence the growth and survival of leukemic cells [14,16,20]. Leukemia associated oncogenic kinases, e.g., Bcr-Abl, Flt3-ITD, c-kit(D816V) or Jak2(V617F) are able to phosphorylate Stat5 and contribute to its persistent activation [15,21,22,23]. Aberrant Stat5 activity has also been observed in solid tumors. Activation occurs mainly as a consequence of mutations or overexpression of cytokine receptors and para- and autocrine signaling of secreted ligands present in the tumor microenvironment. Stat5 activation can cause induction of aggressive tumor cell growth of prostate cancer cells or sustain a more differentiated and non-invasive phenotype in breast tumor cells [17,24].

**Figure 1:**
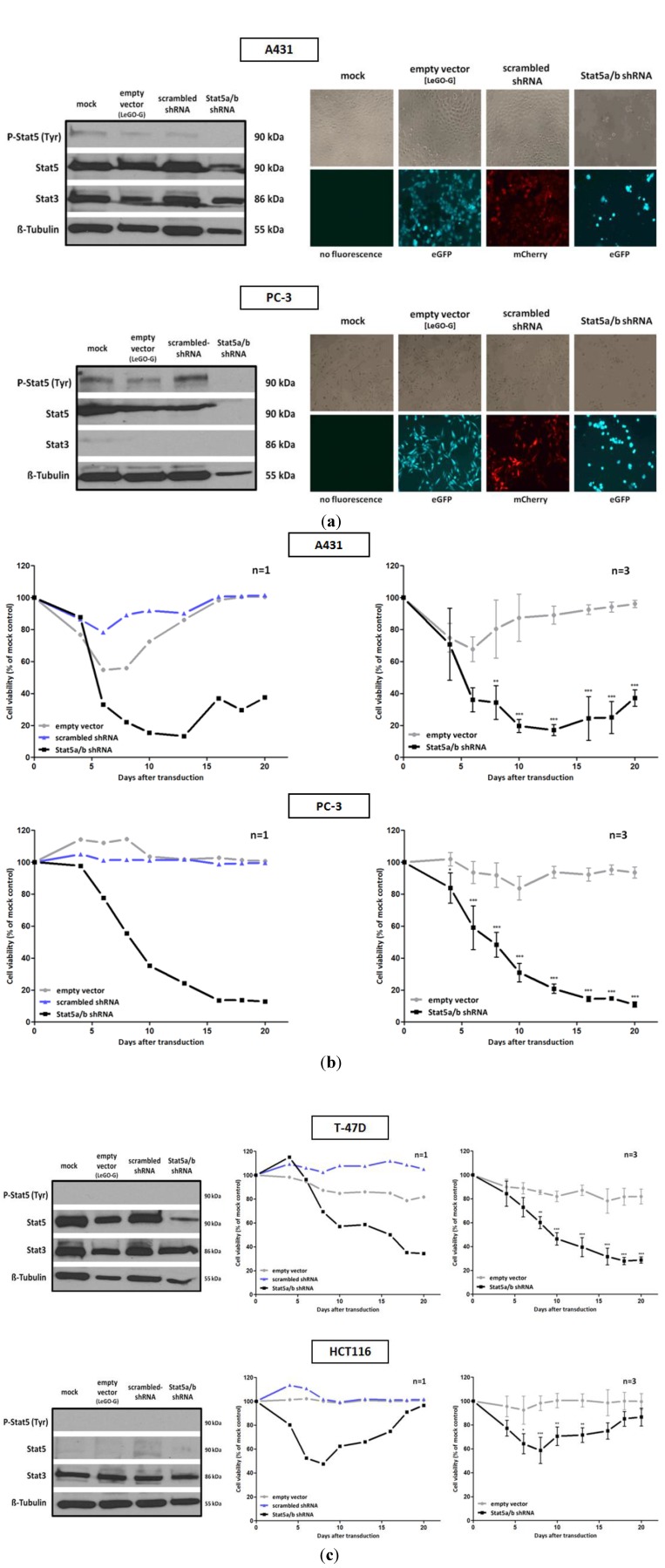
Infection with a lentivirus gene transfer vector encoding Stat5-directed shRNA reduces Stat5 expression and suppresses the growth and viability of human tumor cells. (**a**) A431 epidermal and PC-3 prostate carcinoma cell lines were infected with lentiviruses encoding either the empty vector (LeGO-G: eGFP), a scrambled shRNA (LeGO-C: mCherry) or pairs of shRNA specific for the mRNA of the human Stat5a and Stat5b isoforms.6 days after infection knockdown-efficiency was verified by western blot, using antibodies recognizing either total or tyrosine phosphorylated Stat5 protein. The detection of total Stat3 served as a control of shRNA specificity. Eight days after infection cell density and morphology was documented by phase contrast and fluorescence microscopy. (**b**) Over a period of 20 days after viral transduction changes in proliferation and viability of A431 and PC-3 cells were measured by XTT assay at regular intervals. The infection with empty vector and Stat5-shRNA expressing lentiviruses was done in triplicates and the simultaneous infection with scrambled shRNA expressing lentiviruses was done once. Results are shown as the percentage of viable cells compared to the mock-treated control. (**c**) The same experiments were performed with the T-47D breast and HCT116 colorectal cancer cell line. (n = 3; Ø ± SD) Significantly reduced XTT-values in comparison to empty vector expressing cells are indicated. * *p* < 0.05, ** *p* < 0.01, *** *p* < 0.001 (2-way-ANOVA with Bonferroni correction).

We investigated tumor cell lines derived from different types of human cancer and performed downregulation experiments with Stat5 specific shRNA. The sequences were chosen to affect both isoforms of human Stat5, Stat5a and Stat5b. Four tumor cell lines were selected. They vary with respect to Stat5 expression and activation under normal culture conditions. The prostate epithelial PC-3 cell line, derived from bone marrow metastasis of a patient with grade IV prostate adenocarcinoma, exhibits the highest Stat5 tyrosine phosphorylation when compared to the other cells (Figure 1a). This aggressive tumor cell line is characterized by a deletion of the Stat3 and Stat5b gene locus and exclusively expresses Stat5a [25]. It also carries an amplification of the c-myc gene and p53 and PTEN loss of function mutations [26]. Autocrine prolactin signals (Prl) are responsible for the constitutive activation of Stat5 in these cells, which is further enhanced by reduced levels of SOCS protein (suppressor of cytokine signaling) expression, a negative regulator of Stat signaling [27,28]. The epidermal carcinoma cell line A431 shows a basal level of Stat5 tyrosine phosphorylation, although much lower than PC-3 cells. This cell line is characterized by an overexpression of the prolactin (PrlR) and epidermal growth factor receptor ErbB1. Additionally, the formation of self-activating ErbB2/ErbB4 heterodimers can be detected. Stat proteins, including Stat5, can be found associated with overexpressed ErbB receptors in melanoma and epidermoid carcinoma like the A431 cell line. This association is accompanied by an increased phosphorylation of Stat proteins by the intrinsic ErbB-receptor kinase activity or by recruited Src family kinases [29]. In both cell lines, the downregulation of Stat5 expression by lentiviral transfer of Stat5 directed shRNA, resulted in a strong decrease of cell growth and viability (Figure 1a,b). In the PC-3 cell line, this dependency can possibly be explained to the loss of expression of anti-apoptotic and growth promoting genes, induced by constitutive Stat5a activation. In these cells, it cannot be compensated by Stat3 due the deletion of this gene. In many prostate cancers the transcriptional activity of Stat5 is enhanced by a synergism of active Stat5-dimers with the androgen receptor [30]. Similar effects of Stat5 inhibition by RNAi or antisense oligonucleotides have been observed before [31,32].

The relatively low levels of Stat5 tyrosine phosphorylation in A431 cells suggest that the effects observed upon Stat5 downregulation might be due to Stat5 functions independent from transcriptional induction. In phenotypically related melanoma cells, correlations between the loss of Stat5-mediated expression of the anti-apoptotic genes *Bcl-2* or *Bcl-xL* and enhanced cell death have been observed [33,34]. The delivery of Stat5-shRNA into T-47D breast cancer cells led to a similar conclusion. This cell line has been derived from an invasive adenocarcinoma of the mammary ductal epithelium. It exhibits overexpression of PrlR and the estrogen receptor (ER), but no activated Stat5 was detected under normal cell culture conditions. In spite of the absence of activated Stat5, downregulation of Stat5 by RNAi also resulted in a strong decrease of cellular growth and viability. This indicates a Stat5 dependence of these cells unrelated to its transactivation function (Figure 1c). T-47D cells have been used as a model system to study mechanisms of Stat5-regulated phenotypes. Thereby effects of Stat5 inhibition were analyzed in the context of Prl stimulation. The strong growth suppression, however, induced by the downregulation of Stat5a and Stat5b mRNA utilization, has not been observed before [35,36,37]. The growth inhibition is most likely due to effects resulting from Stat5 downregulation. Cytotoxic effects, caused by an excessive shRNA expression and an oversaturation and exhaustion of the shRNA processing machinery [38] can most likely be excluded. Infection of the cells with viruses encoding an irrelevant shRNA sequence had no influence on the viability of the cells. Since there is little or no Stat5-regulated gene expression in unstimulated T-47D cells, these observations suggest an influence of non-phosphorylated Stat5 on the maintenance of cellular survival.

Recent studies identified non-canonical activities of non-phosphorylated Stat5 involved in the formation of the heterochromatin structure and the function of cell organelles [6,39,40,41]. Non-phosphorylated Stat5 monomers are permanently associated with the Golgi apparatus in endothelial and smooth muscle cells. Downregulation of Stat5a and Stat5b resulted in the dilatation and fragmentation of Golgi cisternae, a tubule-to-cyst change in the ER, the distortion of the nucleus and reduced mitochondrial function. The cells showed a cytoskeletal deformation and a round morphology, comparable to the observations we made with A431 and PC-3 cells (Figure 1a). In contrast, HCT116 colorectal carcinoma cells, characterized by a very low Stat5 expression, survive Stat5 downregulation. The viability of these cells was only slightly influenced by the lentiviral transduction of Stat5-shRNA (Figure 1c). Other Stat family members could possibly compensate for the Stat5 functions. Nevertheless, the morphology of HCT116 cells seemed also slightly to be influenced by Stat5 downregulation. This is reminiscent of recent observations made by conditional Stat5 knockout in the epithelial layer of the intestinal mucosa. It resulted in an increase of NF-κB signaling which affects the permeability of tight junctions [42]. Delayed mucosal wound healing and the loss of intestinal barrier function resulted. This mechanism might also affect HCT116 cells upon shRNA administration. The induction of NF-κB could increase tight junction permeability and a disintegration of the cell mono-layer.

### 2.2. Identification of a Stat5 Specific Peptide Ligand

**Figure 2:**
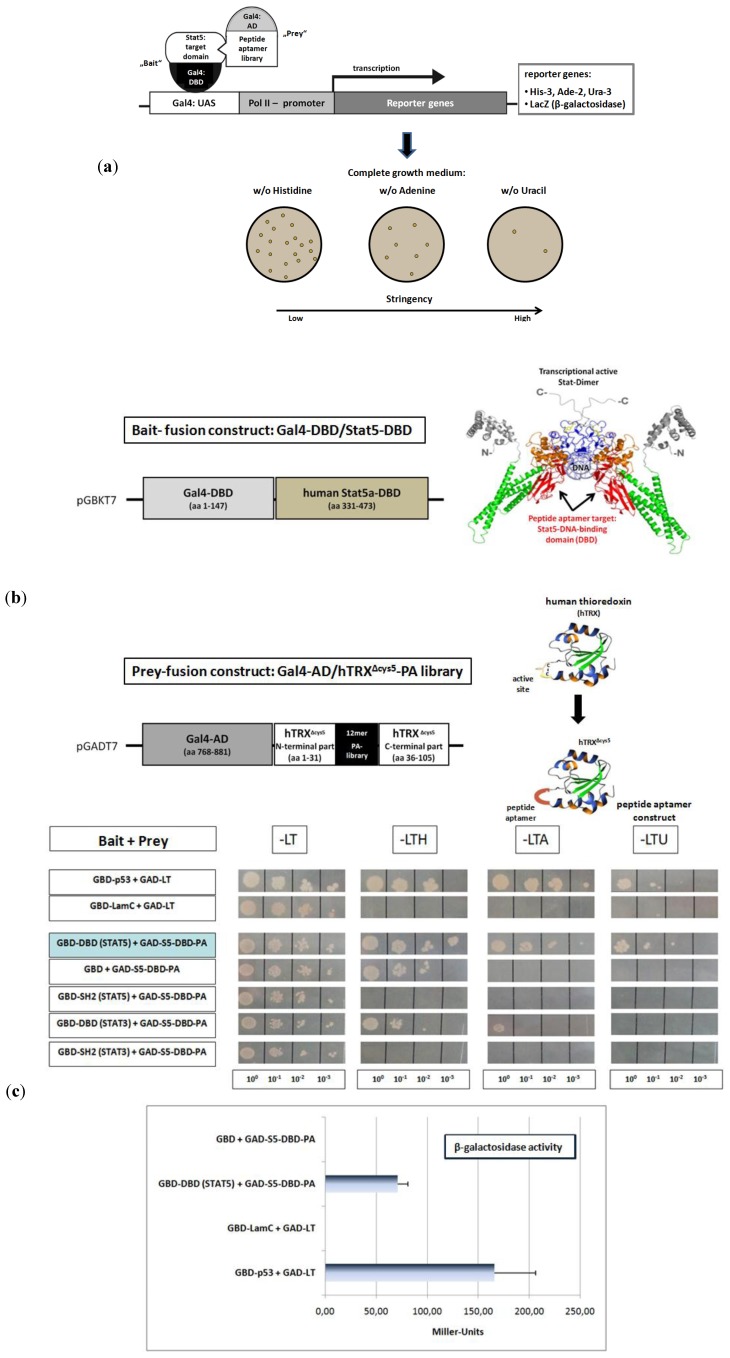
Identification of a 12mer peptide aptamer sequence (S5-DBD-PA) that specifically binds with high affinity to the DNA-binding domain (DBD) of Stat5. (**a**) Yeast-two-hybrid (Y2H) screening strategy for the isolation of PA sequences that interact with a functional domain of human Stat5. Y2H screens were performed with a yeast strain (KF1) containing four different Gal4-dependent reporter genes of different stringency. This included genes for the biosynthesis of histidine (*His-3*), adenine (*Ade-2*) and uracil (*Ura-3*) as well as the *LacZ*-reporter gene for β-galactosidase expression and activity measurement. The corresponding expression vectors (bait and prey) were co-transformed into yeast cells, which were plated and grown under highly stringent conditions on selective media lacking uracil, for identifying the strongest PA interactions. The clone with the best binding properties after revalidation was chosen for further analysis. (**b**) Schematic representation of the cloned bait and prey fusion proteins used for Y2H screening. The DBD of human Stat5A comprises amino acids 331 to 473, indicated in red in the crystallographic structure of a Stat-dimer bound to DNA [47]. This domain was fused to the Gal4 DNA-binding domain (Gal4-DBD) and used as bait. The complex 12-mer PA library was expressed within the active loop of the modified human thioredoxin scaffold (hTRX^Δcys5^, schematically indicated by the hTRX crystal structure), which was fused to the Gal4 transcriptional activation domain (Gal4-AD). These random PA sequences presenting fusion proteins were used as prey constructs.(**c**) The specificity of binding of the identified aptamer (S5-DBD-PA) to the STAT5-DBD was verified after plasmid sequencing and retransformation by plating the yeast cultures in 1:10, 1:100 and 1:1,000 dilutions on selective media lacking leucine (L) and tryptophan (T), which synthesis genes were encoded by the corresponding bait and prey expression vectors, and additionally either histidine (H), adenine (A) or uracil (U). Weak interactions allow growth on -LTH, whereas growth on -LTU requires strong interactions. The interaction of p53 or lamin C with the SV40 Large T antigen served as positive and negative controls. The Gal4-AD (GAD) fused S5-DBD-PA binding properties against the single GAL4-DBD (GBD) and other fused domains of Stat3 and Stat5 (DBD- or SH2-domain) were additionally evaluated. Protein interactions were also quantified by measuring β-galactosidase activity. Results are shown as Miller-Units (n = 3; Ø ± SD).

Peptide aptamer (PA) constructs are fusion proteins composed of a short target specific PA sequence of 10 to 20 amino acids, integrated and presented in a constrained conformation on the surface of a stable scaffold protein. Compared to other combinatorial proteins with therapeutic properties like antibodies or scFv-fragments, they possess several advantageous characteristics. PA are small in size, of simple design and do not contain disulfide bridges. They can be used against intracellular target structures [43,44]. The yeast-two-hybrid system (Y2H) has been established as a suitable method to identify PA sequences able to bind to defined protein target domains [43,45]. This system allows the detection of protein interactions under intracellular conditions in eukaryotic yeast cells. In addition, effects of post-translational folding or secondary modifications, on the ligand protein interactions can be studied. The Y2H system is based on the transcriptional regulation of reporter genes by the dimeric yeast transcription factor Gal4. Gal4 comprises a DNA-binding domain (DBD) and a transcriptional activation domain (AD) and is able to regulate gene expression when both domains are brought into close proximity. This can be accomplished by non-covalent protein interactions, mediated by proteins or protein-domains, expressed as fusion proteins with the Gal4-DBD or Gal4-AD, respectively. Reporter genes with designed, varying promoter strength, can be used to identify protein interactions of different intensity. PA sequences, specific ligands for a pre-determined target domain, can be identified from peptide expression libraries of high complexity. For this purpose, the target protein sequence is fused to the Gal4-DBD, bait fusion protein. The peptides are expressed as fusion proteins with the Gal4-AD, prey fusion proteins (Figure 2a). We used the DNA-binding domain of the human Stat5a protein (Stat5-DBD) as bait (Figure 2b). The DBD of the human Stat5 isoforms exhibit an amino acid sequence homology of 97%. An oligonucleotide library, encoding random 12mer PA sequences and including all amino acids was synthesized. The library has a theoretical diversity of 3 × 10^15^ sequences and a stop codon likelihood of 3.1%. It was prepared for screening as previously described [46].

The peptides were introduced into the protruding, active site of the 12 kDa human thioredoxin protein (hTRX), which served as the PA displaying scaffold fused to the Gal4-AD (Figure 2b). The introduction into the active loop inactivates the enzymatic activity of hTRX. Five cysteine residues present in hTRX, two in the active site and three in the carboxyl terminal part, have been substituted by glycines and serines. This generated an optimized version of the hTRX scaffold (hTRX^Δcys5^), unable to form macromolecular aggregates by disulfide-interactions during protein expression and purification. These modifications strongly favor the monomeric form of recombinant hTRX^Δcys5^ under non reducing conditions [19].

About 1 × 10^7^ distinct constructs from the peptide expression library were transformed into yeast cells and screened as prey-constructs for their binding to the Stat5-DBD under conditions of increasing stringency. Seven yeast clones were isolated. One of them exhibited superior binding strength after re-validation. The affinity of its interaction with the DBD of Stat5 was comparable to the affinity observed for the interaction between p53 and the SV40 largeT antigen (Figure 2c) which was used as a control. The interaction was also confirmed by the measurement of β-galactosidase activity and the Miller units (MU) measured corroborated the intense protein interaction. The specificity of binding of this PA was verified by comparative interaction measurements with the DBD and SH2-domains of Stat3 and Stat5. Only very weak binding to the Stat3-DBD was observed. This reflects the fact that the sequence homology between the DBD of Stat3 and Stat5 is only 42%.

**Figure 3:**
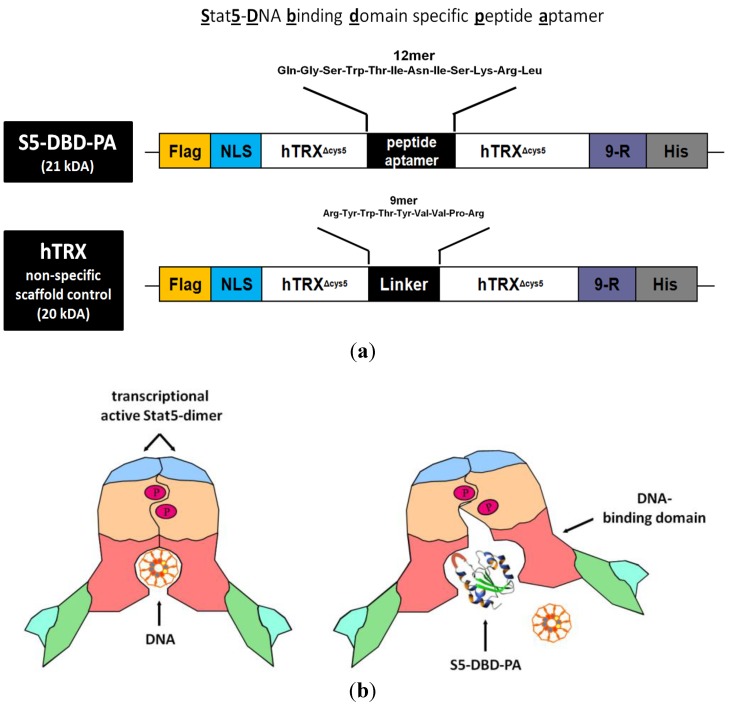
Domain structure of the S5-DBD-PA protein construct used for viral and protein transduction experiments and a model for its inhibitory function. (**a**) The Stat5-DBD specific 12mer PA sequence (S5-DBD-PA) was introduced in the protruding active loop of the hTRX^Δcys5^ protein, which serves as a scaffold for enhanced binding affinity and stability. A protein transduction domain (PTD) consisting of 9 arginines (9-R) is added for intracellular uptake. Additionally a C- terminal histidine tag (His) for purification, a NLS-domain for an enhanced nuclear import and a N-terminal Flag-tag (Flag) for proper detection were added. The complete S5-DBD-PA protein construct has a size of 21 kDa. As a negative control for further analysis the scaffold protein, containing an unspecific 9mer linker sequence instead of the PA sequence was used. This control protein was termed hTRX and has a size of 20 kDa. (**b**) Model for inhibition of the Stat5-DNA-binding activity by the interacting peptide ligand S5-DBD-PA. The specific interaction of S5-DBD-PA with the Stat5-DBD prevents the binding of Stat5 and its DNA response element. It blocks essential regions for the recognition and binding to GAS DNA.

The S5-DBD-PA (Stat5-DBD specific PA) was equipped with functional domains which optimize its recombinant expression, protein purification and delivery into target cells by protein transduction. hTRX^Δcys5^-presented S5-DBD-PA was fused with a protein transduction domain (PTD), consisting of 9-arginine residues (9-R), and with a histidine tag at the carboxyl terminus. A nuclear localization sequence, NLS, for enhanced nuclear translocation and a Flag epitope tag for the immunohistochemical detection upon protein transduction were added to the amino terminus (Figure 3a). This multifunctional framework protein construct has been successfully tested before with a PA of 23 amino acids in length (rS3-PA). This protein is able to specifically interfere with the function of Stat3. It binds to the dimerisation and transactivation domains of Stat3. Recombinant rS3-PA is rapidly taken up by cultured cells upon addition to the growth medium and intracellularly interacts with its target domain. It causes the inhibition of Stat3 activation and dimerisation and reduces the viability and the growth of Stat3-dependent tumor cells [48]. The binding of S5-DBD-PA to the Stat5-DBD is expected to block the function of Stat5 and elicit similar phenotypes as rS3-PA (Figure 3b).

### 2.3. S5-DBD-PA Interferes with the DNA-Binding Activity of Stat5

**Figure 4:**
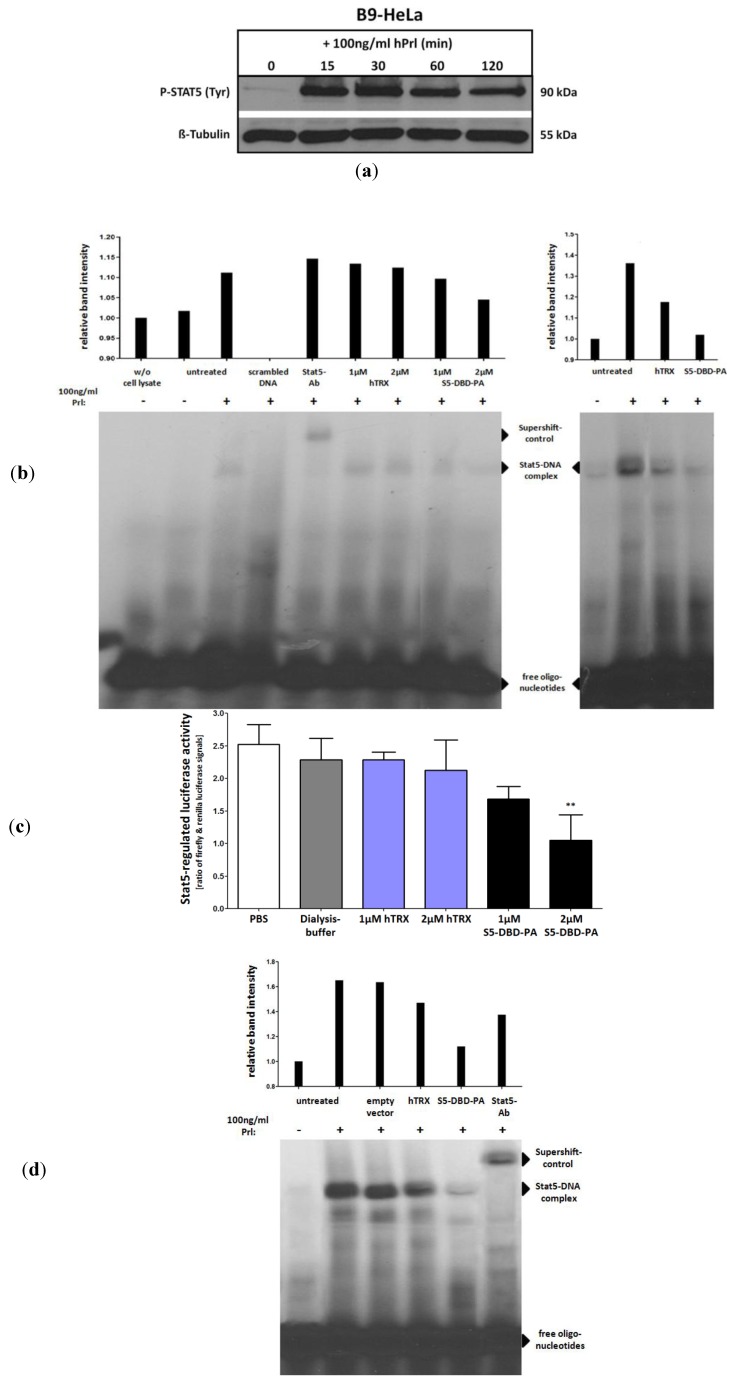
Inhibition of Stat5 DNA-binding by S5-DBD-PA. (**a**) Extracts of modified HeLa cells (B9-HeLa) were used for measuring the Stat5 DNA-binding activity by gelshift and luciferase reporter assays. Tyrosine phosphorylated Stat5-dimers are rapidly induced in these cells by Prl stimulation due to a stable overexpression of Stat5 and the PrlR. (**b**) Evaluation of Stat5 DNA binding activity by electrophoretic mobility shift assay (EMSA). For assessing the therapeutic potential of S5-DBD-PA after being taken up by target cells, Stat5 inducible B9-HeLa cells were treated with recombinant S5-DBD-PA and the non-specific hTRX-scaffold control protein for 4 h in the indicated concentrations (experiment on the left). Stat5 was activated subsequently by adding 100 ng/mL Prl. Untransduced membrane bound proteins were removed by acid-wash prior to lysate preparation. Three µg of the cell lysates were mixed with P^32^-labelled double strand oligonucleotides representing a Stat5 response element of the β-casein promoter. The reaction was carried out at room temperature for 30 min in a binding buffer. Lysates of uninduced or/and untreated cells were used as control. To underline the Stat5 mediated DNA shift a control oligonucleotide and a Stat5 antibody (supershift) was added. Another experiment was done without preincubation of the cells with the recombinant constructs (experiment on the right). B9-HeLa cells were stimulated for 30 min with Prl directly and lysates were prepared. 10 µg of the lysates were incubated for 2 h with 5 µg of recombinant S5-DBD-PA or hTRX under shaking at room temperature. 3 µg of the protein mixtures and 3 µg of untreated B9-HeLa cell lysates (−/+ Prl) were used for the assay. (**c**) Measurement of Stat5-regulated luciferase expression with the dual luciferase reporter assay. B9-HeLa cells were co-transfected with a firefly luciferase Stat5-reporter and a renilla luciferase control reporter to improve the accuracy of the measurement. After 2 days cells were treated for 4 h either with S5-DBD-PA or the scaffold control construct (hTRX) in the indicated concentrations or with the same volume of solvent (dialysis buffer) or PBS. Stat5 was induced by adding 100 ng/mL Prl and luciferase activity was detected after further 4 h. (n = 3; Ø ± SD) Significantly reduced luciferase activities in comparison to PBS treated cells are indicated. ** *p* < 0.01 (1-way-ANOVA with Bonferroni correction) (**d**) Stat5 DNA-binding is inhibited by intracellular S5-DBD-PA expression. STAT5 inducible B9-HeLa cells were transduced with the pSiEW lentiviral vector expressing either S5-DBD-PA, hTRX or the empty vector. 10 days after infection STAT5 was activated by adding 100 ng/mL Prl and cellular lysates were taken. 4µg of the cell lysates were analyzed by EMSA as described before. As controls a Stat5 supershift and lysates of untreated B9-HeLa cells (−/+ Prl) were used.

The PA within the construct S5-DBD-PA was identified on the basis of its affinity to the DBD of Stat5. This binding interaction initially does not ascertain that S5-DBD-PA functions as a Stat5 inhibitor. We carried out experiments which confirmed our expectations that S5-DBD-PA is not only a Stat5-DBD ligand, but also an inhibitor of Stat5 functions. For this purpose electrophoretic mobility shift experiments and promoter-reporter assays were conducted. We observed that S5-DBD-PA can cause the inhibition of Stat5 binding to its DNA response element in gelshift (EMSA) experiments and that it can inhibit a luciferase reporter construct in cells. Recombinant S5-DBD-PA was added to cell culture media or S5-DBD-PA was expressed after lentiviral transduction. The experiments were carried in a HeLa cell line (B9-HeLa), which stably overexpresses Stat5 and the PrlR. The stimulation of these cells with Prl leads to the induction of Stat5 tyrosine phosphorylation mediated by PrlR and Jak2 (Figure 4a). In control experiments, a scaffold construct, hTRX, was used which expresses an irrelevant peptide sequence (Figure 3a).

The application of recombinant S5-DBD-PA to the cell culture medium at concentrations of 1 and 2 µM resulted in the uptake of the protein into the cells and a dose-dependent reduction of Stat5 DNA binding (Figure 4b,c). The recombinant S5-DBD-PA construct also caused the reduction of the expression of the Stat5-regulated luciferase reporter gene. EMSA experiments were also performed with extracts from Prl stimulated, non-transduced B9-Hela cells. The S5-DBD-PA and the hTRX control proteins were added also to cell lysates *in vitro* before the EMSA were carried out, which led to similar effects. The infection of the cells with lentivirus and subsequent intracellular expression of the proteins emphasized the ability of S5-DBD-PA to suppress the complex formation between Stat5 and its DNA response element (Figure 4d).

### 2.4. The Binding of S5-DBD-PA to the DBD of Stat5 Prevents the Nuclear Translocation of Stat5 upon Prolactin Stimulation in T-47D Cells

**Figure 5:**
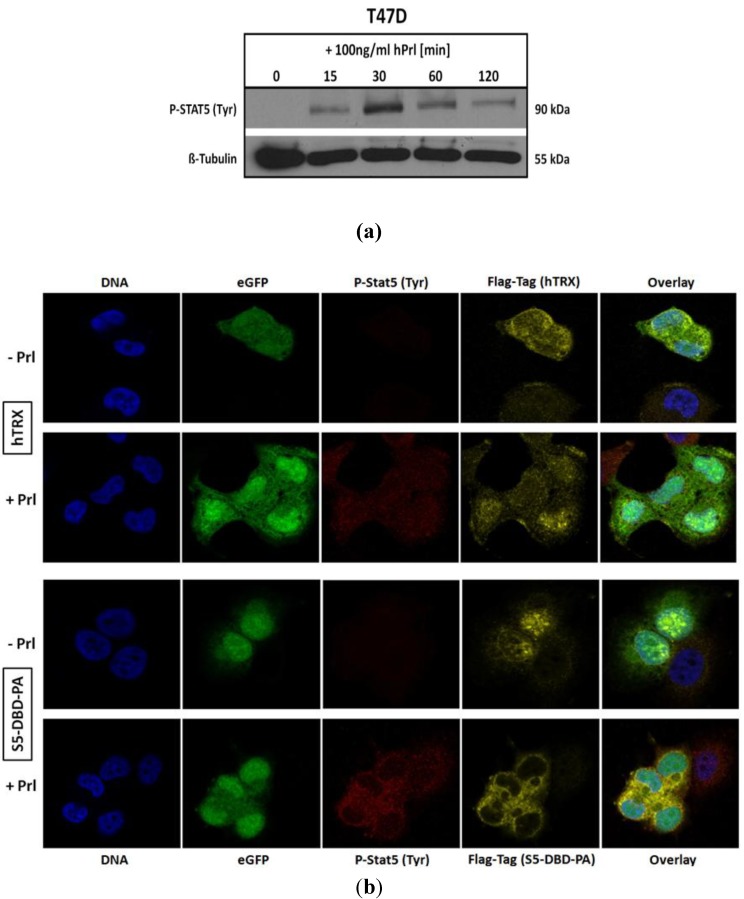
S5-DBD-PA interferes with the nuclear translocation of Stat5 upon prolactin induction. (**a**) Stat5-activation in T-47D breast cancer cells stimulated with Prl. (**b**) Immunofluorescent images of lentiviral transduced T-47D cells either expressing S5-DBD-PA or hTRX were taken 7 days after infection by confocal laser scanning microscopy in the absence or presence of Prl. Cells were stained with a Flag-tag antibody, marked with a Alexa 546 conjugated secondary antibody, and a Alexa 647 conjugated antibody recognizing tyrosine phosphorylated Stat5a and Stat5b. Nuclear staining was performed with DAPI and fluorescence marker (eGFP) expression of the SiEW-lentiviral transfer vector was monitored.

We infected T-47D cells with a lentiviral gene expression vector encoding S5-DBD-PA and analyzed the intracellular location of endogenously expressed S5-DBD-PA by immune fluorescence and confocal microscopy. T-47D cells have been derived from an invasive breast cancer and are characterized by PrlR expression. Stat5 can be activated in these cells by Prl stimulation (Figure 5a). We compared the intracellular location of S5-DBD-PA in Prl treated and control T-47D cells (Figure 5b). In unstimulated T-47D cells, S5-DBD-PA is mainly present in the nucleus. It is possibly associated with unphosphorylated Stat5. Upon treatment of the cells for 30 minutes with Prl, Stat5 was activated and the accumulation of S5-DBD-PA in the cytoplasm was observed. The expression of the hTRX protein was followed in control cells. This protein is equally distributed in cytoplasmic and nuclear cell compartments, independent of a Prl-stimulation. Activated Stat5 enters the nucleus by nuclear translocation, mediated by the interaction of importin-α complexes with a NLS-sequence within the DBD of Stat proteins. They become exposed upon dimer formation [4,49]. In hTRX expressing T-47D cells active Stat5-dimers are found in the cytoplasm and in the nucleus 30 minutes after Prl induction. In S5-DBD-PA expressing cells nearly all tyrosine phosphorylated Stat5 was found in the cytoplasm and co-localized with S5-DBD-PA. We conclude that the binding of S5-DBD-PA to the DBD of activated Stat5-dimers prevents their nuclear translocation, a process which contributes to the functional Stat5 inhibition.

### 2.5. S5-DBD-PA Suppresses the Viability of Tumor Cells

**Figure 6:**
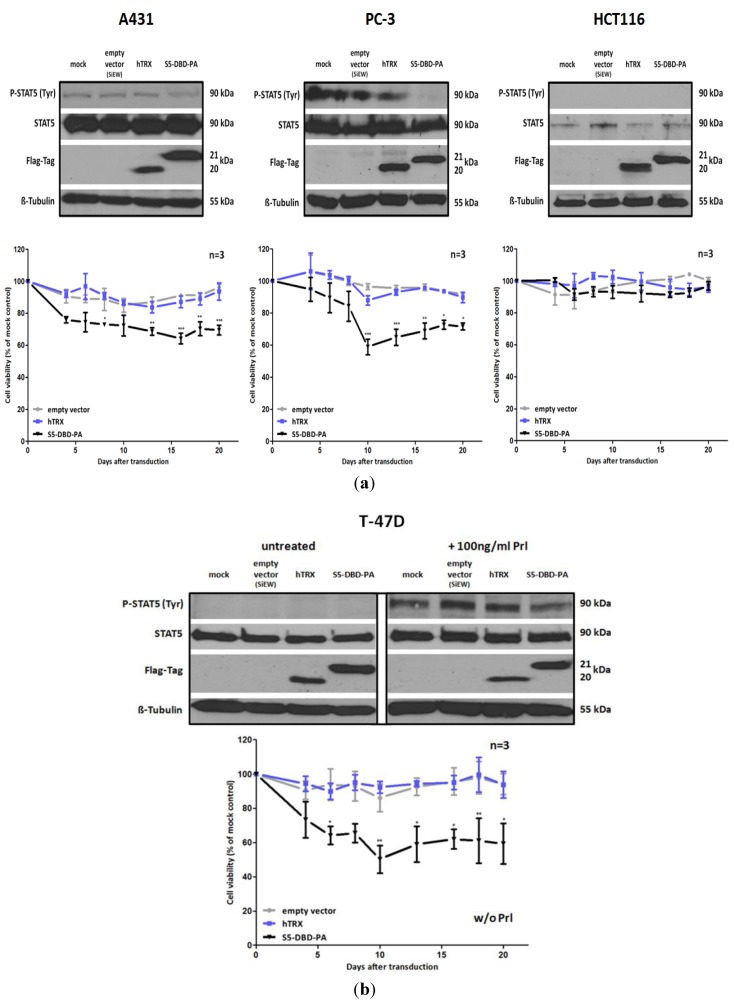
S5-DBD-PA suppresses the viability and the growth of tumor cells. (**a**) A431 epidermal, PC-3 prostate and HCT116 colorectal carcinoma cell lines were infected with SiEW-lentiviral vectors encoding either S5-DBD-PA, hTRX or the empty vector. Over a period of 20 days cell viability and concomitant growth was monitored by XTT assay. Results are shown as the percentage of viable cells compared to the mock-treated control. Stat5 and transgene expression was verified by western blot 7 days after infection using antibodies recognizing either total or tyrosine phosphorylated Stat5 protein and a flag tag antibody. (**b**) The same experiments were done with unstimulatedT-47D breast cancer cells. Protein expression was analyzed after 7 days with and without 30 min Prl stimulation. (n = 3; Ø ± SD). Significantly reduced XTT-values in comparison to empty vector expressing cells are indicated. * *p* < 0.05, ** *p* < 0.01, *** *p* < 0.001 (2-way-ANOVA with Bonferroni correction).

The inhibition of Stat5 function in tumor cells has consequences for cell viability and cell growth. We carried out experiments to evaluate the potential effects of S5-DBD-PA on these parameters. We infected A431, PC-3, T-47D and HCT116 cells with lentiviral vectors encoding S5-DBD-PA, the hTRX non-specific scaffold control or an empty vector. Vector induced expression of S5-DBD-PA resulted in significant reductions of growth and viability during the first three weeks after infection (Figure 6). HCT116 cells, cells with very low Stat5 expression, were only slightly affected. These results confirm the observations made by downregulation of Stat5 expression through RNAi, described above. A431, PC-3 and T-47D cells are most strongly affected. S5-DBD-PA is able to suppress Stat5 function and cause the reduction of cell growth. The growth inhibition induced by a Stat5 mRNA downregulation, however, was more pronounced. It is possible that the shRNA mediated downregulation of Stat5 expression affects more of the Stat5 functions, canonical and non-canonical ones, than the complex formation with S5-DBD-PA. In A431 and PC-3 cells it is reasonable to assign the reduction in cellular viability to the loss of the Stat5 transactivation function due to S5-DBD-PA binding. This confirms previously published results. Prostate cancer cell survival and growth, dependent upon Stat5-regulated gene expression, has been described [24,31]. Not entirely expected, however, was the observation that unstimulated T-47D cells were affected by the S5-DBD-PA ligand. These cells exhibit no Stat5 activation in the absence of cytokine treatment, *i.e.*, their survival is not dependent upon the expression of Stat5 target genes. Immunofluorescence analyses revealed the presence of S5-DBD-PA in the nucleus of unstimulated T-47D cells (Figure 5b). It is possible that S5-DBD-PA is associated with non-phosphorylated Stat5 in the nucleus. This association might inhibit the function of Stat5 as a cofactor of transcription. Such a function has been suggested in support of glucocorticoid receptor mediated transcription in mammary cells or hepatocytes [50,51].

**Figure 7:**
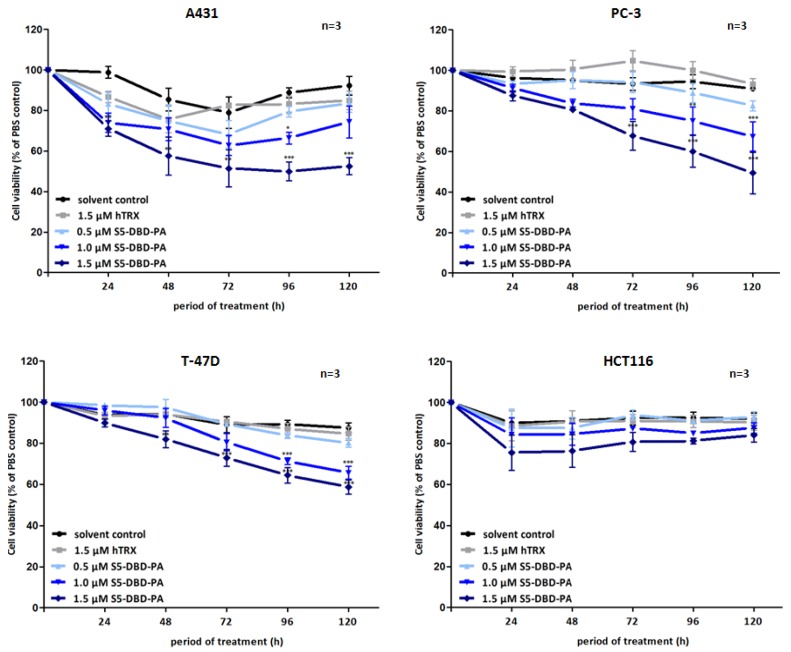
Suppression of tumor cell growth and viability by transduction of recombinant S5-DBD-PA. S5-DBD-PA was transduced into the four tumor celllines A431, PC-3, T-47D and HCT116 by adding 0.5, 1, 1.5 µM of recombinant S5-DBD-PA to the culture medium. The treatment with the scaffold control protein (hTRX) in a 1.5 µM concentration as well as with the same volume of protein-solvent (dialysis buffer) and PBS served as negative controls. Medium and peptides were replaced daily and cell viability and concomitant growth was determined by XTT assay. Results are shown as the percentage of viable cells compared to the PBS control. (n = 3; Ø ± SD) Significantly reduced XTT-values in comparison to protein-solvent treated cells are indicated. * *p* < 0.05, ** *p* < 0.01, *** *p* < 0.001 (2-way-ANOVA with Bonferroni correction).

Non-canonical functions of Stat5 have to be taken into consideration when Stat5 antagonists are being evaluated as potential cancer therapeutics and individual compounds might have differential effects. Tyrosine phosphorylated Stat5 was diminished in the presence of S5-DBD-PA in PC-3 cells and, to lesser extent, also in A431 and Prl stimulated T-47D cells (Figure 6). We do not *a priori* expect that the binding of S5-DBD-PA to the Stat5-DBD should interfere with the phosphorylation of Stat5. This is a consequence we can attribute to the Stat3-specific inhibitor rS3-PA which blocks the phosphorylation site [48]. The reduction of P-Stat5 could be due to enhanced proteasomal degradation of activated Stat5-dimers associated with S5-DBD-PA making S5-DBD-PA a potent inhibitor of Stat5 and Stat5 dependent tumor cells (Figure 7).

## 3. Experimental

### 3.1. Cell Lines and Culture Conditions

The human epidermal carcinoma cell lines A431 (ATCC: CRL-1555) and B9-HeLa (ATCC HeLa: CCL-2) as well as the human prostate cancer cell line PC-3 (ATCC: CRL-1435) and the lentiviral producer cell line 293T (HEK-293T, ATCC: CRL-11268) were grown in DMEM medium. Human HCT116 colorectal cancer cells (ATCC: CCL-247) and T-47D breast cancer cells (ATCC: HTB-133) were cultured in RPMI 1640 medium. Media were obtained from Gibco^®^ Life Technolgies (Carlsbad, CA, USA) or Lonza Group (Basel, Switzerland) and supplemented with 10% FCS, L-glutamine (2 mM) and penicillin/streptomycin (100 U/mL; 100 µg/mL). All cells were passaged regularly every 3–4 days and were grown at 37 °C, 5% CO_2_ and 98% humidity. The modified HeLa cell line B9 was generated before in the lab and is characterized by a stable overexpression of Stat5 and PrlR. For Prl stimulation experiments, 100 ng/mL recombinant human Prl was added to the cell culture medium of B9-HeLa and T-47D cells for 15–30 min.

### 3.2. Reagents

Anti-Flag (M2, clone 2) and -Tubulin (T 0198, clone D66) antibodies were obtained from Sigma-Aldrich (St. Louis, MO, USA). STAT3 (C-20, sc-482) and STAT5 (C-17, sc-835) antibodies were from Santa Cruz Biotechnology (Dallas, TX, USA). P-STAT5 (C11C5, *9359: Tyr694 Stat5a; Tyr699 Stat5b) and P-STAT5-Alexa Fluor^®^647 (C71E5, *9365: Tyr694 Stat5a; Tyr699 Stat5b) antibodies were purchased from Cell Signaling (Cambridge, UK). XTT Cell Proliferation Kit II was obtained from Roche Diagnostics (Rotkreuz, Switzerland). Lipofectamine^®^ LTX & Plus Reagent was from Life Technolgies (Invitrogen, Carlsbad, CA, USA). Recombinant human prolactin (Prl) was purchased from Sigma-Aldrich. Dual-Luciferase^®^ Reporter (DLR™) Assay System was obtained from Promega (Fitchburg, WI, USA).

### 3.3. Plasmid Construction

For interaction assays in the yeast two-hybrid system, pGAD-T7 (prey expression) and pGBK-T7 (bait expression) vectors (Clontech-Takara Bio Europe, Saint-Germain-en-Laye, France) were used. For bait expression the DNA-binding domain (DBD) of the human Stat5a protein (amino acids 331–473) was cloned in frame with the Gal4-DNA binding domain into the MCS of the pGBK-T7 vector. The hTRXΔcys5 scaffold was fused with the Gal4-AD as previously described [19]. The 12mer PA library was synthesized by Eurofins MWG Operon as random oligonucleotides, using a NNK-codon definition (N = A,T,C,G; K = T,G), and with hairpin structure for complementary strand elongation, as described before [46]. By using glycine and proline encoding 5′-*ApaI* and 3′-*SmaI* restriction sites the double strand oligonucleotide-library was cloned into the active site of hTRXΔcys5 in mono-directional NNK order.

For bacterial expression, the pFlag-2 vector (Sigma-Aldrich) was used. In order to create the complete S5-DBD-PA peptide aptamer construct, applicable for protein transduction, purification, nuclear translocation and proper detection, the hTRXΔcys5 scaffold with the Stat5-DBD specific 12mer PA sequence was cloned together with a NLS-sequence, the9-R PTD and the 6xHis tag, C-terminal of the Flag tag as previously described [48].

For the stable expression of human Stat5a and Stat5b directed shRNA the lentiviral pLeGO-G vector (Lentiviral Gene Ontology Vectors: www.lentigo-vectors.de) was used. The validated shRNAs were obtained from Sigma-Aldrich [MISSION^®^ shRNA] introduced into the lentiviral pLKO.1-puro vector with puromycin selectable marker. For the documentation of the transduced cells by fluorescence microscopy and for the determination of precise viral titers by FACS, the shRNA sequences were cloned along with the pLKO.1-puro U6-promoter in the pLeGO-G vector with eGFP selectable marker. Thereby the U6-promoter of the pLeGO-G vector was replaced. The sequences of the processed siRNA antisense strands were for human Stat5a (TRCN0000019308): 5′ ATCCTGAT CGAGTACATGGTC 3′, and for human Stat5b (TRCN0000019356): 5′ ATCTGGCTTGTTAATGA GTAG 3′. A non mammalian shRNA from Sigma-Aldrich [MISSION^®^ shRNA], (*SHC002, TRC1/1.5): 5′ TTGGTGCTCTTCATCTTGTTG 3′, was cloned from pLKO.1-puro into the lentiviral pLeGO-C vector with mCherry red fluorescent marker. For the stable expression of S5-DBD-PA and the hTRX non-specific scaffold control construct in the target cells, the complete Flag-NLS-hTRX-9R-6xHis cassette was cloned into the lentiviral pSiEW vector via a single *SacII*-restriction site. Here transgene expression was driven by a spleen focus-forming virus (SFFV) promoter, which also drives the expression of eGFP through an internal ribosome entry site (IRES). All used lentiviral transfer plasmids had an HIV-1 derived vector backbone with SIN-configuration (self-inactivating) for safety, replication incompetent transgene expression, due to a deletion of promoter and enhancer sequences in the 3′-LTR.

### 3.4. Lentiviral Vector Production and Target Cell Infection

For the infection of the target cells, VSV-G pseudotyped lentiviral SIN-vectors were produced by calcium phosphate coupled transfection of the pCMVΔR8.91 plasmid, encoding HIV-1 derived *gag*, *pol*, *rev* and *tat* genes, the VSV-G envelope encoding pMD2.VSV-G vector and the respective transfer plasmid into the 293T packaging cell line, as described before [52]. Lentiviral particle enriched supernatants were collected 48, 72, and 96 h after transfection, concentrated by ultracentrifugation and stored in aliquots at −80 °C. The viral titer was determined by transducing 293T cells with serially diluted virus, followed by a FACS analysis after 2 days. For transduction experiments, the target cells were infected with an MOI = 20 (multiplicity of infection: amount of viral particles used for infection of one target cell). After 24 h the target cells got fresh media after they were washed with PBS.

### 3.5. Yeast-Two-Hybrid Screen

The PA screening was done in the yeast strain KF1 (MAT_ *trp1–901 leu2–3*, *112 his 3–200 gal4D gal80D LYS2*::*GAL1-HIS3 GAL2-ADE2 met2*::*GAL7-lacZ SPAL10-URA3*) [53], which contains three Gal4-regulated reporter genes for biosynthesis of histidine, adenine and uracil and an additional *LacZ*-reporter gene for β-galactosidase activity measurements. Depending on the promoter strength, these reporter genes allowed a detection of protein interactions with different affinities. As bait, the DNA-binding domain (DBD) of the human Stat5a protein was fused with the Gal4-DNA binding domain, using the pGBK-T7vector as described above [Matchmaker^TM^ GAL4 Two-Hybrid System 3 from Clontech-Takara Bio Europe]. The prey constructs were designed as random 12mer PA expression library with a theoretical diversity of 3 × 10^15^ possible sequences, by using the vector pGAD-T7 [Matchmaker^TM^ GAL4 Two-Hybrid System 3 from Clontech] as template. Therefore the optimized human thioredoxin scaffold (hTRX^Δcys5^) first was cloned in frame with the Gal4-Activation domain, before the synthetic oligonucleotide library was inserted into the active site of hTRX^Δcys5^ (for cloning details see plasmid construction above). Cloned library vectors (prey) were amplified in *E. coli*, from which approximately 1 × 10^7^ different vectors were transformed into yeast and screened. KF1transformants expressing the bait constructs and the prey-library were selected for growth under high stringent conditions in the absence of uracil. Growing KF1-colonies were collected for plasmid extraction and sequence analysis. After that, false positives were excluded and prey-plasmids with correct open reading frame of Gal4-AD, hTRX^Δcys5^ and 12mer PA sequence were retransformed and the interaction was verified by yeast-two-hybrid serial dilution and β-galactosidase assay.

### 3.6. Recombinant Protein Expression, Protein Purification and Transduction

Recombinant expression and subsequent purification of S5-DBD-PA and hTRX by Ni^2+^-chelat affinity chromatography, using a FPLC system, was done as described earlier [19]. For protein transduction of eukaryotic target cells, the recombinant proteins were added to cell culture media in concentrations indicated in the figure legends. To remove untransduced membrane bound proteins, the cells were washed twice with PBS and once with PBS containing 0.2 M acetic acid prior to cell lysis (acid wash).

### 3.7. Electrophoretic Mobility Shift assay (EMSA)

Whole-cell extracts of Prl stimulated B9-HeLa cells, treated with S5-DBD-PA, hTRX or control as indicated in the figure legends, were prepared with a lysis buffer [50 mM Tris (pH 7.4), 100 mM NaCl, 1 mM DTT, 1 mM EDTA, 0.1% Triton X-100, 10% glycerol, phosphatase and protease inhibitors were added freshly] for 30 min on ice. Equal amounts of each extract (3–4 μg total protein) were incubated with radioactive marked oligonucleotides, representing a Stat5-specific response element of the β-casein promoter (5′ TTCTTGGAATTGAAGGGACTT 3′), for 30 min at room temperature in a binding buffer (20 mM HEPES, 1 mM EDTA, 20 mM KCl, 1 mM DTT, 2 mM MgCl_2_, 100 µg/µL BSA). Samples were loaded on a native 4% TBE-polyacrylamide gel and Stat5-DNA complexes were separated from unbound oligonucleotides by electrophoresis. For the verification of the Stat5-DNA complex after electrophoresis a supershift was performed by preincubation of a lysate sample for 20 min with an anti-Stat5 antibody (C17; Santa Cruz Biotechnology) at room temperature. Furthermore a scrambled oligonucleotide and lysates of unstimulated B9-HeLa cells served as control.

### 3.8. Transient Transfection and Dual-Luciferase Reporter Assay

For the measurement of Stat5-regulated luciferase expression prolactin inducible B9-HeLa cells were used and seeded at 0.8 × 10^5^ cells per 24-well. After 24 h the cells were cotransfected with 0.5–1 µg of a firefly-luciferase reporter plasmid (pStat5[RE]_2_-TA-Luc), where firefly luciferase expression is regulated by Stat5 consensus response elements of the β-casein promoter (5′ TTCTTGGAATTGAAGGGACTT 3′), and 25 ng of a constitutively renilla-luciferase expressing control reporter plasmid (pGL4.74[hRluc/TK]). Transient transfection was done with Lipofectamine^®^ LTX (Life Technolgies, Invitrogen) according to the manufacturer’s protocol. After further 24 h the cells were incubated with recombinant S5-DBD-PA, hTRX and control for 4 h, using 2% FCS starvation medium, before Prl stimulation. After another 4 h cells were lysed, detection reagents added and samples measured in a luminometer according to manufacturer’s protocol, using the Dual-Luciferase^®^ Reporter (DLR™) Assay System from Promega. Normalization of the experimental reporter activity against the constitutively expressed renilla-luciferase reference reduced the experimental variability caused by differences in cell viability or transfection efficiency.

### 3.9. Western Blot Analysis

Cells were washed twice with ice-cold PBS and solubilized in immunoprecipitation assay lysis buffer [50 mmol/L Tris (pH 7.4), 150 mmol/L NaCl, 1% NP40, 0.5% sodium desoxycholate, 1 mmol/L EDTA, protease inhibitiors], and incubated on ice for 20 min. Lysates were clarified by centrifugation at 16,000 × *g* for 10 min. For SDS-PAGE, 20–30 µg of each protein sample were loaded. Gels were blotted onto nitrocellulose membranes, which were probed with specific antibodies as indicated in the figure legends. Proteins were visualized with peroxidase-coupled secondary antibodies with the chemiluminescence system (GE-Healthcare, Chalfont St Giles, UK).

### 3.10. Immunofluorescence Staining

Lentiviral transduced and S5-DBD-PA and hTRX expressing T-47D cells were grown on coverslips and stimulated for 30 min with Prl. Cells were fixed and permeabilized with the Cytofix/Cytoperm^TM^ permeabilization and wash buffer system from BD-Becton Dickinson (Franklin Lakes, NJ, USA). For the detection of flag tagged proteins and tyrosine phosphorylated Stat5 the cells were incubated with anti-Flag M2 primary antibody (Sigma-Aldrich) and an Alexa Fluor^®^647 conjugated P-STAT5(Tyr)-antibody (Cell signaling) for 30 min at room temperature with Cytofix/Cytopermwash buffer (antibody amounts were used according to the manufacturer). After the washing steps, an Alexa Fluor^®^546 conjugated anti-mouse secondary antibody (Life Technologies, Invitrogen) was added together with nuclei staining DAPI (1 mg/mL). Cells were incubated for another 30 min. Finally, cells were prepared for confocal laser scanning microscopy by adding mounting medium (ProLong^®^ Gold Antifade; Invitrogen) and analyzed.

### 3.11. XTT - Proliferation and Viability Assay

XTT assays were done to study changes in cell viability and proliferation upon Stat5 inhibition. With lentiviral transduced cells, the experiment was started 24 h after infection by seeding 2,000 cells with 100 µL medium in 96 wells. At intervals of 3 to 4 days the cells were analyzed over a period of 20 days, whereas medium was exchanged regularly. S5-DBD-PA protein transduction experiments were done over 5 days. After seeding again 2.000 cells into 96 wells the experiment was started at the following day. Daily fresh medium was added to the cells, containing either S5-DBD-PA (0.5; 1; 1.5 µM), hTRX (1.5 µM) or the same volume of protein solvent (dialysis buffer) or PBS. The treatment and XTT-measurement was done daily in this experiment. XTT values were measured, according to the manufacturer’s protocol (Roche Diagnostics, Rotkreuz, Switzerland), which assess cell viability via bioreduction of a tetrazolium compound. Spectrophotometric quantification was done at 490 nm on a plate reader.

## 4. Conclusions

Our experiments provide evidence that Stat5 is a promising drug target in cancer therapy and validate peptide ligands as inhibitors of specialized functions of multi-domain proteins. PA can be directed against functional domains of Stat proteins and their complex formation with their intact target can cause the inhibition of phosphorylation, dimerization or DNA binding. These insights can be extrapolated and used in the context of other non-conventional drug targets [54]. Stat5 is expressed in nearly all tissues of mammalian organisms. Stat5 regulated functions comprise the canonical regulation of gene transactivation in the nucleus as well as non-canonical functions in the cytoplasm and mitochondria of cells. The RNAi experiments in this study emphasize the importance of the diversity of Stat5 functions for cellular fates. Downregulation of Stat5 indicates an addiction to Stat5 protein expression in tumor cells in culture. Several recent reports indicate that Stat5 inhibition could become beneficial for patients with varying indications. The JAK1/2 inhibitor ruxolitinib has been used to prevent Stat5 activation in patients with myeloproliferative neoplasms (MPN). It blocked cytokine and inflammatory responses and caused the cell cycle arrest of MPN cells, the cells did not show a reduction in Stat5 expression or the induction of apoptosis. The systemic interference with Stat5 activation, however, might also be associated with side effects. Persistent ruxolitinib treatment caused viral infections due to detrimental effects on immune cell functions [55]. Stat5 ligands which only affect discreet functional domains of Stat5 might become useful to avoid unwanted side effects. The inhibition of particular, tumor cell specific functions of Stat5 would be desirable and peptide based ligands could function as lead compounds [56,57]. Advances in delivery methods could result in the improvement of drug like properties of these peptides. Alternatively, their introduction into appropriate screening systems might yield low molecular weight compounds with similar functional properties [54].
